# JCHLA/JABSC editorial policies and practices to address systemic racism and improve equity, diversity, and inclusion

**DOI:** 10.29173/jchla29554

**Published:** 2021-04-02

**Authors:** Sandra McKeown, Alanna Campbell, Amanda Caputo, Rachel Couban, Colleen Pawliuk

**Affiliations:** 1 Health Sciences Librarian, Queen’s University, Kingston, ON; 2 Public Services Librarian, Northern Ontario School of Medicine, Sudbury, ON; 3 Sault Area Hospital, Sault Ste. Marie, ON; 4 McMaster University, Hamilton, ON; 5 Department of Pediatrics, BC Children's Hospital, Vancouver, BC

In the December 1^st^, 2020 issue of JCHLA/JABSC, the editorial team committed to finding meaningful ways to give Black, Indigenous, and People of Colour (BIPOC) more visibility, and to promote equity, diversity, and inclusion (EDI) [1]. To that end, we pledged to encourage and seek out diverse and inclusive representation when recruiting prospective volunteers for the editorial team; invited submissions about racial injustice and topics of equity, diversity, and inclusion within the scope of health sciences librarianship; and requested feedback from our readership about other ways our Journal can address racism and improve EDI. We renew this commitment for 2021, and want to update you about our position, going forward.

Shortly after the December 2020 issue was published, the editorial team became aware of a specific incident of anti-Black racism occurring in our discipline. Briefly, an invited editorial on anti-Blackness in medical librarianship for the Journal of the Medical Library Association (JMLA) was withdrawn by its five Black librarian authors before publication over challenges and barriers experienced during the editorial process. (A preprint version of the editorial is available via DigitalCommons@UNMC [2].) In mid-December, some of the editorial’s authors shared their experience online [3-5]. Shortly thereafter, the JMLA editor-in-chief issued a public apology taking responsibility for the journal’s failure to properly handle the editorial on anti-Blackness in libraries and outlined steps that will be taken to better prepare JMLA to publish these types of articles [6].

JCHLA sincerely laments that the voices of the five Black authors were not honoured and respected during the JMLA editorial process, and for the pain and frustration this must have caused. We commend the authors for acting and leading with conviction amidst pressure from members of the MLA community, and for sharing their experiences publicly so that this unfortunate series of events can be an opportunity to learn and do better.

An *Open Call for JMLA and Medical Librarianship to Support Black Colleagues* was circulated around the same time as the JMLA apology, demanding a list of actions to be undertaken by the JMLA editorial board and MLA staff in order to foster an equitable publishing environment for Black librarians [7]. JMLA has since responded to the *Open Call* [8] but this case study on anti-Black publishing practices provides a learning opportunity for other journals to carefully consider their own policies and practice, including JCHLA/JABSC. The experience of these authors and the resulting list of actions demanded in the *Open Call* demonstrate that there is much more work to be done for journals like ours to address systemic bias in the publishing process. To this end, the JCHLA/JABSC editorial team met with members of the CHLA/ABSC Board and members of the CHLA/ABSC Diversity, Equity & Inclusion Task Force in January to discuss what happened and additional actions that the Journal might take to address racism and improve EDI. Our editorial team then met separately to review the actions demanded in the *Open Call* to determine how we can start working towards the implementation of these actions over the short and long term.

Moving forward, JCHLA/JABSC will be instituting the following courses of action:

- **Mandating anti-racism and/or EDI professional development for all members of the editorial team:** the editorial team will have *at least* one meeting every year to discuss professional development activities (recently completed individually and/or as a group) on topics of anti-racism and/or EDI. The first meeting (of at least one per yearly term) is to take place in the fall to coincide with the start dates of new position terms.
- **Diversifying the Journal’s editorial team:** calls for new editors will ask applicants to include an EDI statement about relevant experience/training with the intent to diversify the editorial team, or to at least recruit editorial members who can demonstrate they are committed to these efforts.
- **Recruiting individuals with appropriate expertise to serve as peer reviewers:** calls or invitations for peer review will recruit individuals with expertise in critical race theory, gender and sexuality studies, ethnic studies, etc. to serve as peer reviewers or third-party reviewers for submissions on these topics.

Some quick and straightforward actions that the editorial team have already incorporated include:

- **Providing author guidelines for bias-free language**: author instructions under *Preparation of the Manuscript* on the Journal’s website now state that authors must strive to use language that is free of bias and avoid perpetuating prejudicial beliefs or demeaning attitudes in their writing. Authors are encouraged to consult the APA guidelines and recommendations available for Bias-Free Language [9].
- **Encouraging journal submissions on topics of anti-racism and EDI:** the list of suggested topics on the *About the Journal* section of the JCHLA/JABSC website now includes: “Racial injustice and topics of equity, diversity, and inclusion pertaining to health sciences librarianship.” Calls for submissions to the journal will specifically suggest these topics more frequently.

The Journal’s editorial team will continue going through the list of actions demanded in the *Open Call* in order to identify and implement all actions that are applicable for JCHLA/JABSC. While well-intentioned, we acknowledge that there may be missteps along the way and encourage our readership, the medical librarianship community, and beyond, to keep us accountable as we continue this journey of growing, learning, and unlearning so that JCHLA/JABSC can serve our BIPOC colleagues, and by extension the medical librarianship profession, better. We recognize that this work will be on-going and will strive to build continuity amidst turnover of editorial team members. Moving forward it will be imperative for the editorial team to communicate the progress of this work with our readership to facilitate accountability, transparency, and on-going feedback.

The significant list of actions demanded in the *Open Call for JMLA and Medical Librarianship to Support Black Colleagues* is a stark reminder of how far we still have to go in efforts to address racism and improve EDI in scholarly publishing practices. We must move beyond complacency and good intentions to meaningful actions in the fight against systemic racism in scholarly publishing, as in all areas of life.
